# Length-free near infrared measurement of newborn malnutrition

**DOI:** 10.1038/srep36052

**Published:** 2016-11-08

**Authors:** Fatin Hamimi Mustafa, Emily J. Bek, Jacqueline Huvanandana, Peter W. Jones, Angela E. Carberry, Heather E. Jeffery, Craig T. Jin, Alistair L. McEwan

**Affiliations:** 1 School of Electrical and Information Engineering, Faculty of Engineering and Information Technologies, University of Sydney, New South Wales, Australia; 2 Sydney School of Public Health, University of Sydney, New South Wales, Australia; 3 Former Senior Staff Specialist in Neonatology, Royal Prince Alfred Hospital, Sydney, Australia

## Abstract

Under-nutrition in neonates can cause immediate mortality, impaired cognitive development and early onset adult disease. Body fat percentage measured using air-displacement-plethysmography has been found to better indicate under-nutrition than conventional birth weight percentiles. However, air-displacement-plethysmography equipment is expensive and non-portable, so is not suited for use in developing communities where the burden is often the greatest. We proposed a new body fat measurement technique using a length-free model with near-infrared spectroscopy measurements on a single site of the body - the thigh. To remove the need for length measurement, we developed a model with five discrete wavelengths and a sex parameter. The model was developed using air-displacement-plethysmography measurements in 52 neonates within 48 hours of birth. We identified instrumentation required in a low-cost LED-based screening device and incorporated a receptor device that can increase the amount of light collected. This near-infrared method may be suitable as a low cost screening tool for detecting body fat levels and monitoring nutritional interventions for malnutrition in neonates and young children in resource-constrained communities.

Accurate determination of the nutritional status of newborns is a major public health problem because under-nutrition increases the risk of immediate mortality and impacts on growth and cognitive development^1^. According to the World Health Organization (WHO), 44% of all under-five child deaths every year are neonates in their first 28 days of life, with most of these deaths occurring in the first week of life and the greatest incidence of these occurring in low-middle income settings^2^. Accurate assessment of body composition is vital as early identification of under and over nutrition in neonates and children and can guide interventions for nutritional management for clinicians and health-care workers^3^. Conventional approaches for recognising under-nutrition include the use of population-based percentiles (<10^th^, 5^th^, or 3^rd^ percentiles), which rely on weight for gestational age and sex^4^. An alternative to the weight percentiles is to measure body composition.

A variety of methods are available to assess body composition in neonates and children. One high-cost technique uses the PEA POD system, a method based on air displacement plethysmography (ADP). The ADP technique (PEA POD; COSMED, Concord, CA) is often considered the criterion method for determining body composition and is accurate, safe, and noninvasive^5^. Carberry *et al*. showed that under-nutrition and risk of neonatal morbidity are more closely associated with body fat percentage (BF%) measured using ADP rather than the conventional birth weight percentiles^4^. Another high cost and highly accurate technique is dual-energy X-ray (DEXA), however its use is limited to one scan per year as it uses low dose ionising radiation^6^. Deuterium dilution for the measurement of total body water may be an ideal method for newborns as it involves less compliance, but requires trained staff for accurate dose delivery and sample collection, and carries a risk of delay due to time requirements for sample processing. Other techniques such as hydrostatic underwater weighing are unsuitable for newborns while less expensive techniques such as a skinfold thickness measurements require a high degree of training and can be inaccurate with poor predictive value due to incorrect lifting of the skin fold during the measurement, especially in lean newborns^7^. In this light, there is an urgent need for low cost and portable devices to assess body composition and nutrition as a point of care tool.

A relatively new technique for measuring body fat levels in neonates and children uses the near-infrared (NIR) interactance method. The NIR interactance method studies the response of light at specific wavelengths to variations in thickness of the subcutaneous fat layer. The principle is based on light interaction with the various tissue types including the skin, muscle, bone and fat. Depending on the wavelength and optical properties of each tissue, the light is absorbed or reflected by different magnitudes before being captured by a photo-detector^8^. NIR is considered safe, rapid and noninvasive if the power of the incident light is low enough not to heat the skin. This method can also be made mobile and affordable, where a NIR device can be directly connected and easily monitored via portable computing devices. However, the NIR method is limited by its sensitivity to hydration and skin color^8^,^9^. NIR body fat has been studied extensively in the adult population^8^,^10^,^11^,^12^, but there is limited research on its use in neonates and young children^13^. Studies have found that healthy neonates and adults possess different skin structures including thickness of skin layers, size of cells and size of fibres, where the parameters are smaller in neonates^14^,^15^,^16^. Neonatal skin appeared to be more hydrated than adult skin under an electron micrograph^14^,^16^ and, in general, water content in newborns was higher than in adults at 81% compared to 73%^17^.

Previously, we found that NIR measurements taken on the newborn’s thigh combined with weight and length data can provide a reasonable estimate for BF%^18^. The device was based on inexpensive LEDs and photodiodes. In this paper we consider a reflectance NIR measurement system having two different configurations: with and without a cosine corrector device connected at the collecting side of the probe. The cosine corrector acts as an optical diffuser that allows light to be collected from a wider range of angles compared with capturing the light using an uncorrected sub-miniature A-type (SMA) fibre cable.

We have developed a statistical model to estimate BF% in newborns from NIR reflectance measurements based on BF% from ADP measurements. As hydration is the major concern affecting NIR measurements in newborns, we exploit the NIR spectral absorption peaks of fat and water. Our model utilises three different ratios using five different wavelengths and an additional sex parameter. Our aim is to develop the best model for NIR measurement based on the ADP measurements, not to compare the two measurement methods at this stage.

## Methods

### Data Collection

The NIR measurements were taken at the tertiary referral hospital, Royal Prince Alfred Hospital (RPAH), Sydney between September 2014 and December 2014 on newborn of various ethnic backgrounds. Maternal conditions during pregnancy, birth details and maternal and paternal demographics including ethnicity, age, height, weight, date of birth, and education background were recorded. The measurements of all subjects were conducted in duplicate or triplicate on the skin surface of both anterior and medial thighs. The thigh was chosen as the measurement site because it is a convenient location that is accessible while breastfeeding. All devices were tested for medical safety by the RPAH Biomedical Engineering department and were found to meet IEC60601 medical safety regulations.

### Measurement Set up

**Figure 1:**
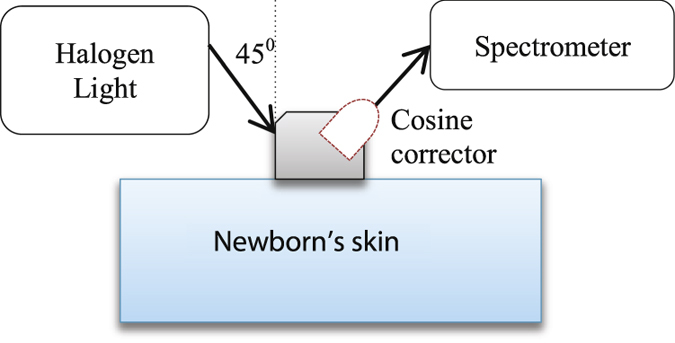
Near-infrared body fat measurement set up. Dotted object is the cosine corrector.

A schematic of the NIR measurement setup is illustrated in Fig. 1. The cosine corrector device is shown with a dotted line to indicate that it may or may not be included in the measurement process. A tungsten halogen light (Mikropack HL-2000-FHSA, 6.7 mW, 360 nm to 2400 nm range) was connected to a 3D-printed fibres holder via a SMA fibre (Thorlabs, M28L01, ∅400 μm, 0.39 *NA*). The holder was designed with two holes positioned at −45° and +45° angles to the normal surface to locate the transmitter and receptor fibres respectively. At the receptor side, another SMA fibre (Thorlabs, M14L01, ∅50 μm, 0.22 *NA*) was used to optically connect the holder to a spectrometer (Ocean Optics QEPRO-FL, 350 nm to 1100 nm range, SNR 1000:1) and then the response signal was recorded for 20s with OceanView 1.4 software (Ocean Optics). The measurement was repeated on different subjects with a cosine corrector (Thorlabs CCSA1, ∅4 mm) coupled between the holder and the SMA fibre receptor.

BF% and weight were recorded by the PEA POD ADP measurement system. The BF% was determined by placing the naked newborn inside a closed chamber and air displacement was measured using pressure and volume changes. Body density was calculated from measured body mass and the calculated body volume^19^. Gestational age and length were obtained from the PEA POD database for data analysis.

### Diffuse Reflectance Model

A simplified diffuse reflectance model of a layered medium, *R*(*x*, *y*) from the Radiative Transfer Equation (RTE) with a corrected diffuse approximation (CDA) was defined^20^:


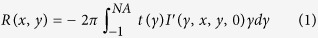


where *NA* is defined as the numerical aperture of the detector used that is aligned normally to the boundary plane at *z* = 0, the *t*(*γ*) is the Fresnel transmission coefficient due to the refractive index mismatch at the boundary, and the quantity *I’* relates to *I* as:


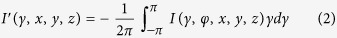


where *I*(*γ*, *φ*, *x*, *y*, *z*) is the radiance over the range of angles (*γ* = cosine *ϑ*, and *φ*) exiting the skin collected by the detector at positions indicated by the vector *<x*, *y*, *z>*. The angle *ϑ* is the elevation angle with respect to the *z-axis* in spherical coordinates, while *φ* is the azimuthal angle of the position vector. Note that the range of −*π* < *φ* *<* *π* is due to the assumption of uniform scattering. The *I*(*γ*, *φ*, *x*, *y*, *z*) depends on the optical properties: the absorption coefficient as function of absorption length, *μ*_*a*_ (*l*_*a*_), the scattering coefficient as function of scattering length, *μ*_*s*_ (*l*_*s*_), and the anisotropy, *g.* In detail these are given by:


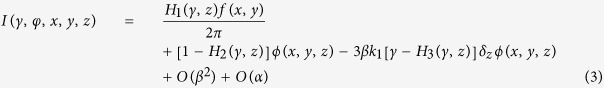


where *β* = *1/w*(*μ*_*s*_), *α* = *μ*_*a*_/*μ*_*s*_, *k*_*1*_ = *1/3μ*_*s*_(*1* − *g*). The *w* is the beam width, the *ϕ* (*x*, *y*, *z*) is the solution of the boundary value that can be solved either by Laplace’s equation or the diffusion equation. Meanwhile, the *f*(*x*, *y*) is the incident beam profile, which is set as Gaussian beam, and *H*_*n*_ (*n* = *1*, *2*, *3*) denotes a half space Green’s function^20^. Whilst the *t*(*γ*) from equation (1) is given by:


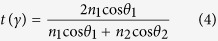


where *n*_*1*_ and *n*_*2*_ are the refractive indices of the ambient material and skin respectively, *θ*_*1*_ is the transmission angle of the light source and *θ*_*2*_ is the reflected angle of the light from the skin. The *NA* from equation (1) is given by:


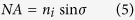


where *n*_*i*_ denotes the refractive index of material outside the fibre, which in our case is the air (*n* = 1), and *σ* donates maximum half acceptance angle of the fibre or cosine corrector. The *NA* of the used cosine corrector is 1.0 compared to 0.22 of the SMA fibre as stated in section 2.2, which result in acceptance angles from 0° to 180° and from 77.3° to 102.7° for the cosine corrector and the SMA fibre cable respectively.

### Statistical Model Development

**Figure 2:**
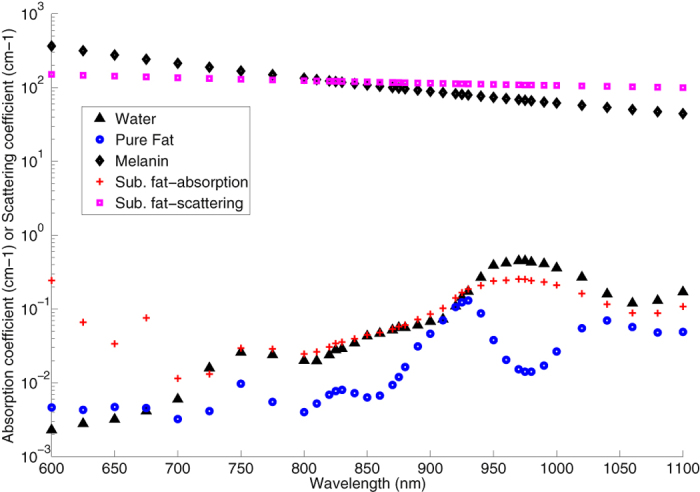
Absorption coefficient spectrum of melanin, absorption coefficient spectrum of pure water, absorption coefficient spectrum of pure fat and absorption coefficient spectrum of subcutaneous fat layer and scattering coefficient spectrum of subcutaneous fat layer.

In previous NIR studies, the absorbance (A) ratio at two different wavelengths, *A*(*λ*_*1*_)/*A*(*λ*_*2*_) was derived by K. Norris *et al*. in order to remove and normalise the baseline offset^21^. The idea of a ratio at two different wavelengths was used in developing our statistical model. To reduce the influence of water absorption, our preference was given to select ratios that were based on wavelengths highly influenced by fat and water^9^,^22^,^23^. Figure 2 shows the scattering spectrum of the subcutaneous fat layer, absorption spectrum of pure fat, absorption spectrum of melanin, absorption spectrum of pure water and also calculated absorption spectrum of subcutaneous fat layer following the Meglinski’s equation model. From Fig. 2, the dominant effect of water can be observed between 850 nm and 1050 nm. The spectral curve of the subcutaneous fat layer imitates the curve of pure water even though the peak of pure fat is clearly at 930 nm. This is due to the water content in the subcutaneous fat layer^9^. Scattering has a high influence in the subcutaneous fat layer (in Fig. 2), but the unidentified fat and water constituents over the spectrum make them difficult to separate.

We planned to use more than two wavelengths that were highly influenced by fat and water to counter the effect of melanin in the epidermal layer^9^, shown in Fig. 2. As a consequence of the high absorption possessed by the melanin spectrum, we only selected subjects from the white skin category in developing our statistical model due to very low number of subjects from the other skin categories.

We used Matlab software (Version R2012b; Mathworks Incorporated, Natick, Massachusetts), SPSS (Version 22), Microsoft Excel (Version 2013) and R (Version 3.3.1) to perform the statistical analysis. Linear piecewise interpolation was first applied on the spectrometer readings to determine the reflection in 10 nm intervals within the range of 850 nm to 1100 nm. The model development process involved evaluating all possible ratios of these readings to determine key combinations of wavelengths that exhibited the highest correlation with BF% measured by ADP. A maximum of three sets of ratios and sex were used as input variables for an ordinary least-squares linear regression model, with NIR BF% as the target variable and BF% from ADP as a reference. Model performance was evaluated on the basis of coefficients between the predicted and actual values (significant level of 0.05, two-tailed test). The prediction of BF% by NIR was evaluated against ADP BF% from regression lines. The distribution of the data was tested using a Shapiro-Wilk test to ensure that the differences were normally distributed (Gaussian). Residuals were also plotted to compare variability of the developed model over the range of ADP measurements.

### Ethics

This study was approved by the Human Research Ethics Committee of the Royal Prince Alfred Hospital, Sydney, Australia and all experimental methods were carried out in accordance with relevant guidelines and regulations (Protocol No.; X14-118, HREC/09/RPAH/645). Informed and written consent was obtained from the parents of the newborns.

## Results

**Figure 3:**
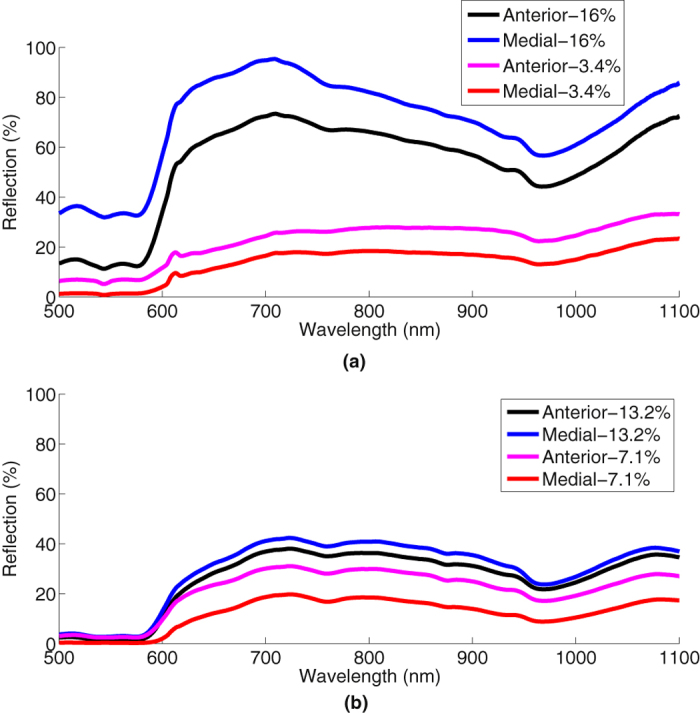
Near-infrared reflection from the anterior and medial thighs of the highest and lowest BF% of two subjects in each cohort (**a**) with cosine corrector, (**b**) without cosine corrector.

Sixty subjects were first measured using ADP. They were then split into two cohorts: Cohort 1 was measured using the NIR device with a cosine corrector fitted (the first 30 subjects) and Cohort 2 was measured without a cosine corrector (the next 30 subjects). To mitigate the risk of excessive movement, the newborns were measured while sleeping, immediately after a feed or during feeding. Figure 3(a,b) show NIR reflection spectra obtained from NIR measurements with using the cosine corrector and without using the cosine corrector respectively both for the anterior and medial thighs of two subjects. The subject selection was based on those having the highest and the lowest BF% from the ADP measurements. At this level of detail there appeared to be baseline offsets of the spectra that supported the implementation of ratios at two different wavelengths in the developed model.

Table 1 shows the characteristics of the neonates studied (total n = 60: n = 30 for each cohort). The development of the model in this study only considered white skin subjects (total n = 52: n = 26 for each cohort) due to the high influence of melanin over NIR spectra. The data for dark skin subjects could not be meaningfully modeled due to low numbers (total n = 8: n = 4 for each cohort). We found significant correlation between NIR absorption using the cosine corrector and ADP for both anterior and medial thighs of white skin subjects (correlation coefficient, R = 0.877 and R = 0.839 respectively) as shown in Table 2. Root Mean Squared Error (RMSE) and *p*-values of Cohort 1 (white skin subset) of both sites are generally lower than those of Cohort 2. This is attributed to the greater light detection obtained using the cosine corrector.

**Figure 4:**
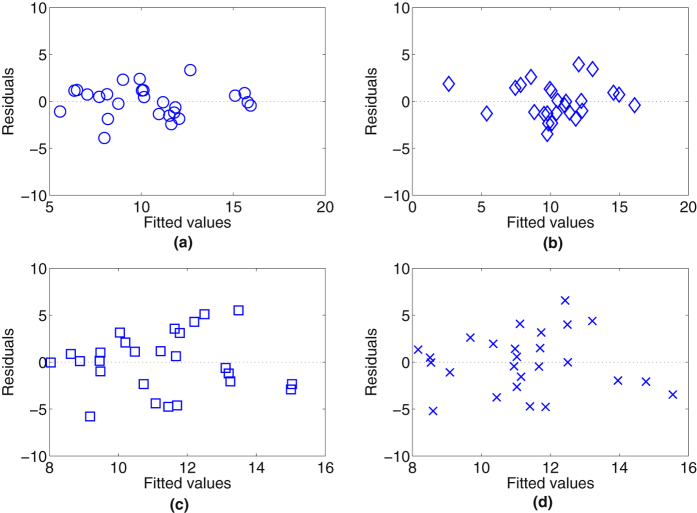
Residual plots of NIR BF% and ADP BF% with white skin subjects (total n = 52: n = 26 for each cohort). Plot (**a**) NIR with cosine corrector on anterior thigh, (**b**) NIR with cosine corrector on medial thigh, (**c**) NIR without cosine corrector on anterior thigh while (**d**) NIR without cosine corrector on medial thigh.

The residual plots shown in Fig. 4 with and without the cosine corrector for both anterior and medial thighs of white skin subjects are evenly and are randomly dispersed throughout the x-axis (fitted value), which indicates that the assumptions of linearity and homoscedasticity are valid. The Shapiro-Wilk test also shows that the data sets follow a normal distribution.

The model based on white skin subjects utilised three different reflection ratios at five different wavelengths and a parameter of sex as:


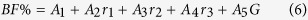


where BF% is the body fat percentage measured using ADP, *r*_*1*_ is the ratio of 890 nm/1020 nm, *r*_*2*_ is the ratio of 920 nm/1010 nm, *r*_*3*_ is the ratio of 1010 nm/900 nm, and *G* is the sex which is assigned the value of 1 for male and 0 for female. *A*_*1*_, *A*_*2*_, *A*_*3*_, *A*_*4*_, and *A*_*5*_ indicate the constant coefficients of the respective parameter. The values of these constant coefficients were obtained following a statistical model and are shown in Table 3.

Illustrated in Table 4 are the mean and standard deviation of the NIR BF% obtained from equation (6) and the ADP BF% of the two cohorts for the anterior and medial thighs of white skin subjects. Notice that the mean value for ADP BF% and NIR BF% are completely in agreement. The variability of the measurements is less for NIR (2.83%) than ADP (3.62%). The residual plots show a dissimilarity of variability between cohorts, where the residual of the NIR with the cosine corrector (Fig. 4(a,b) for anterior and medial thighs respectively) was lower than the NIR without using the cosine corrector (Fig. 4(c,d) for anterior and medial thighs respectively) demonstrating less variability with the cosine corrector.

**Figure 5:**
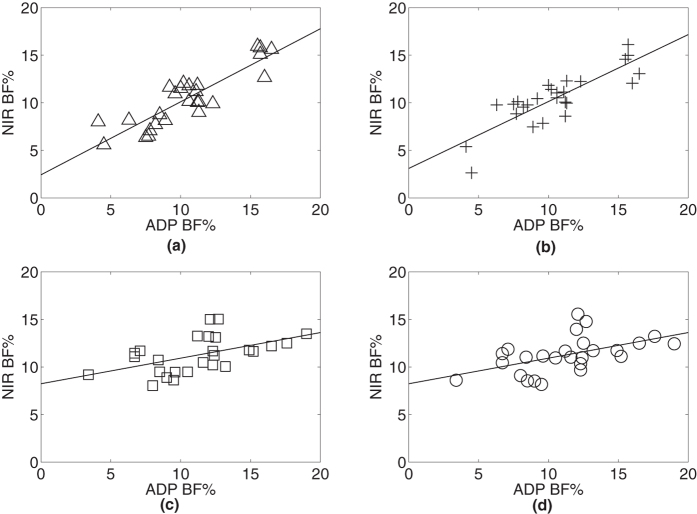
Linear regression lines of NIR BF% and ADP BF% with white skin subjects (total n = 52: n = 26 for each cohort). Plot (**a**) NIR with cosine corrector on anterior thigh, **(b**) NIR with cosine corrector on medial thigh, (**c**) NIR without cosine corrector on anterior thigh while (**d**) NIR without cosine corrector on medial thigh.

The regression lines of the NIR model BF% against the ADP BF% show that the NIR model over-estimates BF% for lean neonates, with the cosine corrector (Fig. 5(a,b)) reducing the over-estimation of BF% compared to the model without the cosine corrector (Fig. 5(c,d)).

Melanin or skin colour has been found to influence NIR measurements on skin^9^. We extended our analysis to include all subjects of both cohorts (n = 60: n = 30 for each cohort) for anterior and medial thighs using our developed NIR BF% model. The effect of including the mixed skin colours in the analysis is reflected in the statistical results in Table 5, which shows reduced correlation values and higher RMSE and *p*-values than those presented in Table 4. Nevertheless, the statistical results in Table 5 (mixed skin colour) are consistent with the results in Table 4 (white skin colour only) in showing that NIR using the cosine corrector was always better than NIR without using the cosine corrector.

As the choice of using three different wavelength ratios was somewhat arbitrary, we show the effects on R using between one and five ratios in our NIR BF% model for white skin colour subjects in Table 6 (n = 26 of cohort 1 for both anterior and medial thighs). The selection of wavelengths was still referred to absorption peaks of water and fat. The increases of R which resulted from increasing the number of wavelength ratios used agrees with a study of breast imaging by Lo *et al*., which they found that adding more wavelengths up to eight improved extraction errors^24^. Although the R continues to increase with the number of wavelength ratios used, the increase is leveling off and the inclusion of these additional wavelengths would involve more cost in the final device which is intended for use with discrete diodes for low cost. We included the subject length parameter in Table 6, row 5 for comparison with other independent ratios of NIR where it can be seen that it provides at least as much information as two additional ratios or three additional wavelengths.

## Discussion

To our knowledge, this is the first study developing NIR BF% for neonates using BF% from a gold standard body composition ADP technique to develop models. Our findings showed that NIR configuration with the cosine corrector resulted in the highest correlation and the lowest residual. We found that the NIR measurements after adding the cosine corrector showed higher correlation between NIR BF% and ADP BF% (R = 0.877 and R = 0.839 for anterior and medial thighs respectively on white skin subjects). The NIR using the cosine corrector also showed the lowest residuals with the ADP BF% indicating the ability to fit an improved model. The improvement offered by the cosine corrector in the NIR body fat measurement demonstrates the importance of appropriate equipment and device selection in NIR configurations. This was in agreement with a study by Hwang *et al*., in which they used varied types of LEDs with different view angles (lamp at 20° over miniature chip at 120°) in NIR phantom experiments resulting in higher sensitivity by the miniature chip^25^.

Anthropometric parameters (e.g.; length and weight) and age were not included in our developed model as these measurements are often unknown or inaccurate in low resource settings. Our objective was to determine whether this NIR technique could be used independently for easy point of care measurement of nutritional status. Our developed model however depended on an additional parameter, sex, because this factor influences BF% at birth^26^. Nevertheless, the sex parameter is always accessible and does not need any equipment. Measurement of length has been used as one of the primary indicators of foetal, neonatal and child nutrition^27^. However, length measurements are often problematic due to inter- and intra-observer variability unless appropriate equipment including an appropriate length board with extensive training are used^27^. The Futrex device requires anthropometric parameters of age, weight, height and level of exercise in their developed model^28^. Those parameters may often be inaccessible or unreliable, especially in a low-income setting.

Past NIR subcutaneous fat studies using simulations and phantom measurement methods have found that light reflection increases logarithmically with the thickness of the fat layer^25^,^29^. The logarithmic relationship can be attributed to the high influence of fat on light scattering. As the thickness of fat increases, more light is scattered back to the ambient surface until at a critical thickness, the point where the light is saturated and there is no increase in backscattered light with any further increases of the thickness of fat. This critical thickness constrains the NIR method to the lower subcutaneous fat thicknesses associated with undernutrition in neonates and children. For example the subcutaneous fat layer beneath the thigh skin of low birth weight and normal full term neonates were found to be 1.7 mm to 3.0 mm and 3.0 mm to 5.0 mm respectively^12^. To our knowledge, there have been no specific studies to determine NIR maximum thickness detection, which might rely on the type of NIR devices used and acceptance power source emitted^25^.

The strengths of our study include that this was the first development of NIR BF% models using BF% from ADP as a reference in a newborn population in order to look at the potential of direct and low cost LED-based NIR device being comparable to the gold standard ADP in screening undernutrition and morbidity in newborns. NIR commercial devices, such as Futrex have only targeted on children above 5 years to adult populations^30^,^31^,^32^. Since this study was only conducted in newborns, other populations across infancy (newborn up to 2 years) with higher number of subjects, different skin colours and environments (temperatures) need to be investigated in order to establish the developed model and to formally test levels of agreement on a different dataset than that used to develop the models. Testing for agreement with several other gold standard body composition techniques using new NIR dataset is also essential to ensure the robustness of the NIR BF% model. For implementation, five LEDs could be allocated around a low-cost cosine corrector that couples to a wavelength-range detector and filters.

In conclusion, we have developed a NIR-based BF% model using gold standard ADP measurements. The developed NIR BF% models have utilised three ratios at five different wavelengths with the introduced cosine corrector to determine newborn body composition. The results showed significant correlation and agreement with ADP. We have shown that our device may have the potential to identify undernourished newborns who are at significant risk of associated morbidity such as hypothermia, hypoglycaemia and mortality in settings where a gold standard device would not normally be available. This is particularly useful for low resource settings where equipment to screen for hypoglycemia (glucometers, blood glucose analysis) and hypothermia (low reading thermometers) are limited or lacking. In such settings, a point of care, accurate, robust and low-cost device is needed to distinguish between the pathologically versus constitutionally small for gestational age neonates. The future plan is to test the device in a randomised controlled trial measuring relevant health outcomes.

## Additional Information

**How to cite this article**: Mustafa, F. H. *et al*. Length-free near infrared measurement of newborn malnutrition. *Sci. Rep.* **6**, 36052; doi: 10.1038/srep36052 (2016).

**Publisher’s note:** Springer Nature remains neutral with regard to jurisdictional claims in published maps and institutional affiliations.

## Figures and Tables

**Table 1:** Characteristics of 60 subjects studied.

	White Skin*	Dark Skin*	Total	Sex	Body Fat % (Mean ± SD)	Gestational Age *Weeks* (Mean ± SD)	Weight *Kg* (Mean ± SD)	Length *cm* (Mean ± SD)
Cohort 1 With cosine corrector	26 (Caucasian-23 (88%)), Asian-3 (12%))	4 (Asian-4 (100%))	30	63% Male	10.10 ± 3.41	39.28 ± 1.60	3.23 ± 0.49	49.57 ± 3.18
Cohort 2 Without cosine corrector	26 (Caucasian-16 (62%), Asian-10 (38%))	4 (Asian-3 (75%), Aboriginal-1(25%))	30	50% Male	11.13 ± 3.72	39.47 ± 1.27	3.31 ± 0.43	49.42 ± 2.19

^*^Based on ethnicity information with skin colour recorded.

**Table 2:** Results from statistical analysis of NIR absorption for anterior and medial thighs of white skin subjects (total n = 52: n = 26 for each cohort).

	Anterior thigh			Medial thigh		
	R	RMSE	*P*-value	R	RMSE	*P*-value
Cohort 1 (white skin subset, n = 26)	0.877	1.77	<0.001	0.839	2.0	<0.001
Cohort 2 (white skin subset, n = 26)	0.519	3.38	0.143	0.519	3.38	0.143

**Table 3:** Values of the constant coefficients used in equation (6).

	A_1_	A_2_	A_3_	A_4_	A_5_
Cohort 1 (white skin subset)					
Anterior thigh	−317.70	255.18	−83.25	193.38	−1.64
Medial thigh	−186.79	225.77	−141.47	115.04	−1.81
Cohort 2 (white skin subset)					
Anterior thigh	−214.29	103.11	−0.64	128.45	2.26
Medial thigh	−225.26	111.74	1.27	127.77	−2.07

**Table 4:** Mean and standard deviation of NIR BF% in equation (6) and ADP BF% of two cohorts of white skin subjects (total n = 52: n = 26 for each cohort).

	ADP BF% (Mean ± SD) %	NIR BF% (Mean ± SD) %	
		Anterior thigh	Medial thigh
Cohort 1 (white skin subset, n = 26)	10.44 ± 3.373	10.44 ± 2.832	10.44 ± 2.830
Cohort 2 (white skin subset, n = 26)	11.26 ± 3.617	11.26 ± 1.875	11.26 ± 1.876

**Table 5:** Results from statistical analysis of NIR absorption for anterior and medial thighs of all subjects (total n = 60: n = 30 for each cohort) with (Cohort 1) and without (Cohort 2) a cosine corrector.

	Anterior thigh			Medial thigh		
	R	RMSE	*P*-value	R	RMSE	*P*-value
Cohort 1 (all subjects, n = 30)	0.820	2.10	<0.001	0.719	2.53	<0.001
Cohort 2 (all subjects, n = 30)	0.363	3.74	0.45	0.420	3.64	0.28

**Table 6:** BF% estimations, R and RMSE of less and more than three ratios.

No.	Cohort 1 – With Cosine Corrector (n = 26)	R/RMSE (Anterior thigh)	R/RMSE (Medial thigh)
1	*BF%* = *r*_*1*_ + *G*	0.498/3.05	0.462/3.12
2	*BF%* = *r*_*1*_ + *r*_*2*_ + *G*	0.602/2.87	0.721/2.49
3	*BF%* = *r*_*1*_ + *r*_*2*_ + *r*_*3*_ + *G* + ***r***_***4***_	0.877/1.81	0.839/2.05
4	*BF%* = *r*_*1*_ + *r*_*2*_ + *r*_*3*_ + *G* + ***r***_***4***_ + ***r***_***5***_	0.879/1.84	0.854/2.01
5	*BF%* = *r*_***1***_ + *r*_***2***_ + *r*_***3***_ + *G* + ***L***	0.891/1.71	0.867/1.88

Ratios of *r*_*1*_, *r*_*2*_, and *r*_*3*_ are the ratios in equation (6) while added ratios of *r*_*4*_, and *r*_*5*_ are 930 nm/1050 nm, and 970 nm/930 nm respectively and *L* is length. Subjects are from white skin colour (n = 26 for Cohort 1).

## References

[b1] Levitsky D. A. & Strupp B. J. Malnutrition and the brain: changing concepts, changing concerns. The Journal of nutrition 125, 2212S–2220S (1995). 7542703 10.1093/jn/125.suppl_8.2212S

[b2] Organization W. H. World health statistics 2010. (World Health Organization, 2010).

[b3] Wells J. & Fewtrell M. Measuring body composition. Archives of disease in childhood 91, 612–617 (2006). 16790722 10.1136/adc.2005.085522 PMC2082845

[b4] Carberry A. E., Raynes-Greenow C. H., Turner R. M., Askie L. M. & Jeffery H. E. Is body fat percentage a better measure of undernutrition in newborns than birth weight percentiles? Pediatric research 74, 730–736 (2013). 24002331 10.1038/pr.2013.156

[b5] Christensen K. & Kushner R. Measuring body fat in the clinical setting. Obesity Management 3, 93–95 (2007).

[b6] Damilakis J., Adams J. E., Guglielmi G. & Link T. M. Radiation exposure in X-ray-based imaging techniques used in osteoporosis. European radiology 20, 2707–2714 (2010). 20559834 10.1007/s00330-010-1845-0 PMC2948153

[b7] Olhager E. & Forsum E. Assessment of total body fat using the skinfold technique in full‐term and preterm infants. Acta Paediatrica 95, 21–28 (2006). 16373292 10.1080/08035250500323731

[b8] Conway J. M., Norris K. H. & Bodwell C. A new approach for the estimation of body composition: infrared interactance. The American journal of clinical nutrition 40, 1123–1130 (1984). 6507337 10.1093/ajcn/40.6.1123

[b9] Jacques S. L. Optical properties of biological tissues: a review. Physics in medicine and biology 58, R37 (2013). 23666068 10.1088/0031-9155/58/11/R37

[b10] Möller R. . Estimating percentage total body fat and determining subcutaneous adipose tissue distribution with a new noninvasive optical device LIPOMETER. American Journal of Human Biology 12, 221–230 (2000). 11534019 10.1002/(SICI)1520-6300(200003/04)12:2<221::AID-AJHB8>3.0.CO;2-2

[b11] Hong H. K., Jo Y. C., Choi Y. S., Park H. D. & Kim B. J. An optical system to measure the thickness of the subcutaneous adipose tissue layer. Paper presented at *The 8th Annual IEEE Conference on Sensors: IEEE SENSORS 2009*, *Christchurch*, *New Zealand*. San Diego, CA: IEEE, (doi: 10.1109/icsens.2009.5398349) (Oct., 2009).

[b12] Hwang I. D. & Shin K. Fat thickness measurement using optical technique with miniaturized chip LEDs: A preliminary human study. Paper presented at *Engineering in Medicine and Biology Society*, *2007: 29th Annual International Conference of the IEEE*, IEEE, (10.1109/iembs.2007.4353351) (2007). 18003017

[b13] Kasa N. & Heinonen K. Near‐infrared interactance in assessing superficial body fat in exclusively breast‐fed, full‐term neonates. Acta Pædiatrica 82, 1–5 (1993). 10.1111/j.1651-2227.1993.tb12504.x 8453201

[b14] Holbrook K. A histological comparison of infant and adult skin. Neonatal Skin: Structure and Function. New York: Marcel Dekker, 3–31 (1982).

[b15] Barel A. O., Paye M. & Maibach H. I. Handbook of cosmetic science and technology. (CRC Press, 2014).

[b16] Stamatas G. N., Nikolovski J., Luedtke M. A., Kollias N. & Wiegand B. C. Infant skin microstructure assessed *in vivo* differs from adult skin in organization and at the cellular level. Pediatric dermatology 27, 125–131 (2010). 19804498 10.1111/j.1525-1470.2009.00973.x

[b17] Wang Z. . Hydration of fat-free body mass: new physiological modeling approach. American Journal of Physiology-Endocrinology and Metabolism 276, E995–E1003 (1999). 10.1152/ajpendo.1999.276.6.E995 10362610

[b18] McEwan A. . Low-cost near-infrared measurement of subcutaneous fat for newborn malnutrition. Paper presented at SPIE Smart Structures and Materials+ Nondestructive Evaluation and Health Monitoring, International Society for Optics and Photonics. (10.1117/12.2044764) (2014).

[b19] Ward L. C., Poston L., Godfrey K. M. & Koletzko B. Assessing Early Growth and Adiposity: Report from an EarlyNutrition Academy Workshop. Annals of Nutrition and Metabolism 63, 120–130, 10.1159/000350702 (2013). 23969405

[b20] Rohde S. B. Modeling diffuse reflectance measurements of light scattered by layered tissues, PhD thesis, University of California (2014).

[b21] Workman Jr J. & Springsteen A. Applied spectroscopy: a compact reference for practitioners. (Academic Press, 1998).

[b22] Meglinski I. V. & Matcher S. J. Quantitative assessment of skin layers absorption and skin reflectance spectra simulation in the visible and near-infrared spectral regions. Physiological measurement 23, 741 (2002). 12450273 10.1088/0967-3334/23/4/312

[b23] Vogel A. J. Noninvasive Optical Imaging Techniques as a Quantitative Analysis of Kaposi’s Sarcoma Skin Lesions, PhD thesis, University of Maryland (2007).

[b24] Lo J. Y. . Wavelength Optimization for Quantitative Spectral Imaging of Breast Tumor Margins. PLoS ONE 8, e61767, 10.1371/journal.pone.0061767 (2013). 23613927 PMC3629043

[b25] Hwang I. D., Shin K., Ho D.-S. & Kim B.-M. Evaluation of chip LED sensor module for fat thickness measurement using tissue phantoms. Paper presented at *Engineering in Medicine and Biology Society*, *2006: 28th Annual International Conference of the IEEE*, IEEE. (10.1109/iembs.2006.259965) (2006). 17946731

[b26] Hawkes C. P. . Gender-and gestational age–specific body fat percentage at birth. Pediatrics 128, e645–e651 (2011). 21824882 10.1542/peds.2010-3856

[b27] Wood A. J., Raynes-Greenow C. H., Carberry A. E. & Jeffery H. E. Neonatal length inaccuracies in clinical practice and related percentile discrepancies detected by a simple length-board, Neonatal length measurement inaccuracies. Journal of paediatrics and child health 49, 199–203, 10.1111/jpc.12119 (2013). 23432733

[b28] Hartmann S. . Phantom of Human Adipose Tissue and Studies of Light Propagation and Light Absorption for Parameterization and Evaluation of Noninvasive Optical Fat Measuring Devices. Optics and Photonics Journal 5, 33 (2015).

[b29] Nilubol C., Treerattrakoon K. & Mohammed W. S. Monte Carlo modeling (MCML) of light propagation in skin layers for detection of fat thickness. Paper presented at *Southeast Asian International Advances in Micro/Nano-technology*, International Society for Optics and Photonics. (10.1117/12.863536) (2010).

[b30] Fthenakis Z. G., Balaska D. & Zafiropulos V. Uncovering the FUTREX-6100XL prediction equation for the percentage body fat. Journal of medical engineering & technology 36, 351–357 (2012). 22953764 10.3109/03091902.2012.708382

[b31] Heyward V. H. & Gibson A. Advanced fitness assessment and exercise prescription 7th edition. (Human kinetics, 2014).

[b32] Moon J. R. . Percent body fat estimations in college women using field and laboratory methods: a three-compartment model approach. Journal of the International Society of Sports Nutrition 4, 16 (2007). 17988393 10.1186/1550-2783-4-16 PMC2212632

